# Does the Progression of the COVID-19 Pandemic Have an Influence on the Mental Health and Well-Being of Young People? A Cross-Sectional Multicenter Study

**DOI:** 10.3390/ijerph182312795

**Published:** 2021-12-04

**Authors:** Zeliha Özlü-Erkilic, Oswald D. Kothgassner, Thomas Wenzel, Andreas Goreis, Anthony Chen, Veysi Ceri, Aylin Fakhr Mousawi, Türkan Akkaya-Kalayci

**Affiliations:** 1 Outpatient Clinic of Transcultural Psychiatry and Migration Induced Disorders in Childhood and Adolescence, Department of Child and Adolescent Psychiatry, Medical University of Vienna, Währinger Gürtel 18-20, 1090 Vienna, Austria; zeliha@gmx.at; 2 Postgraduate University Program Transcultural Medicine and Diversity Care, Medical University of Vienna, Spitalgasse 23, 1090 Vienna, Austria; Aylin-Mousawi@gmx.at; 3 Department of Child and Adolescent Psychiatry, Medical University of Vienna, Währinger Gürtel 18-20, 1090 Vienna, Austria; oswald.kothgassner@meduniwien.ac.at; 4 Department of Psychiatry and Psychotherapy, Medical University of Vienna, Währinger Gürtel 18-20, 1090 Vienna, Austria; thomas.wenzel@meduniwien.ac.at; 5 Scientific Section on Psychological Aspects of Torture and Persecution, World Psychiatric Association (WPA), 1226 Thônex, Switzerland; anthonyfchen@gmail.com; 6 Department of Clinical and Health Psychology, Faculty of Psychology, University of Vienna, 1010 Vienna, Austria; andreas.goreis@univie.ac.at; 7 Outpatient Unit for Research, Teaching and Practice, Faculty of Psychology, University of Vienna, 1010 Vienna, Austria; 8 Department of Child Development, Faculty of Health Sciences, Batman University, Merkez Kampüsü, Batman 72060, Turkey; veysiceri@gmail.com

**Keywords:** COVID-19 pandemic, mental health, psychological well-being, young people, Austria, Turkey

## Abstract

The COVID-19 pandemic has been shown to have impaired the mental health and well-being of young people. This study, for the first time, explores these aspects in young people with and without a migratory background during the extended course of the pandemic and restrictive measures, comparing two countries with a high COVID-19 prevalence: Austria and Turkey. Methods: The authors used the “Psychological General Well-being” index as part of an anonymous online survey with 3665 participants (ages 15–25), recruited from both countries during the first and the second waves of the pandemic, collecting data on individual experiences and problems encountered during the pandemic. Results: Mental health (b = 0.06, *p* < 0.023) and general psychological well-being worsened with the progression of the pandemic. Participants with financial problems had the most severe negative effect on mental health (b = 0.12, *p* < 0.001). Furthermore, females living in Turkey, both natives (b = −0.21, *p* < 0.001) and migrants (b = 0.25, *p* < 0.001), reported a more deteriorated mental health status over time. Conclusions: The extended pandemic duration and resultant “lockdown” restrictions have negatively affected the mental health of young people to varying degrees, depending on country of residence and migration background. A strong “recovery plan” that considers group-specific needs and vulnerabilities is urgently needed.

## 1. Introduction

In early 2020, the World Health Organization (WHO) confirmed the first COVID-19 wave to be a pandemic [1]. This first wave spread rapidly around the world, reaching all continents within only a few months and affecting many countries in a very short time [2].

Globally, during the initial phase of the pandemic, the infection and mortality rate for minors with COVID-19 were initially low, compared with the adult population. In the later stages of the pandemic, these rates rose continuously, especially as vaccination rates are lower in some groups of minors [3,4]. Similar to other countries, both Austria and Turkey later decided to implement different forms of restrictions (“lockdowns”) on the population’s interactions and movement, with the goal of slowing or stopping the COVID-19 pandemic. Restrictions such as school closures or curfew could be expected to cause some distress among children and young adults, leading to personal crises [5,6] but might affect different groups to different degrees.

Around the world, more than 1.5 billion youths, which is about 90% of the world’s students, were banned from schools during the initial lockdowns [7]. School closures may be especially difficult for youths, as social contact plays an important role in their psychological development and well-being. Therefore, pandemic restrictions may have a negative impact on the well-being of younger individuals [7,8]. During the pandemic, psychological stress, violence, aggression, and poverty increased in many families due to a number of factors [9,10,11], potentially including the need for home-schooling [7]. Furthermore, the pandemic has led to a severe reduction in the provision of child mental health care, child welfare, and child protection resources [9]. A number of studies consequently reported that psychological problems among children and adolescents have increased during the COVID-19 pandemic [12,13,14]. During the Ebola epidemic in West Africa, the rate of child labor, sexual abuse, neglect, and pregnancies among adolescents was relatively high, indicating that pandemics might create severe risks besides the deterioration of mental health for children [15].

Globally, the COVID-19 pandemic and its adverse outcomes have impaired the mental health [16] and well-being [17] of many individuals. However, diverse studies have reported that the threat of infection with COVID-19 and its critical outcomes have affected minority groups and migrants more than a country’s majority native population [18,19]. As migrants most commonly work as front-line employees in many countries, they may have greater exposure to COVID-19 infection and its adverse consequences [20], in addition to the specific impact of lock-down-based restrictions on these groups. Several studies have consequently demonstrated the severe impact of the pandemic and pandemic-related measures specifically on the mental health and well-being of migrant populations [21,22,23,24,25,26,27].

### Aims of the Study

The purpose of this study is to examine and compare the situations of migrant and non-migrant groups in two countries with high COVID-19 prevalence, different local cultures (individualistic or collectivistic), and the psychological impact of the pandemic’s associated control measures. We also wanted to explore the evolution of these aspects over time during the multiple waves of the pandemics, and the resultant stress on younger population groups.

## 2. Material and Methods

An anonymous online survey (SoSci Survey) was administered to adolescents and young adults at two time points corresponding to the first two “waves” of infection: first from 22 May to 19 June and then from 11 September to 23 October 2020, in two countries with different pandemic control strategies and cultures but with high COVID prevalence: Austria and Turkey. Subjects were recruited via different social media channels and by reaching out to institutions such as youth centers, which work with adolescents and young adults. The inclusion criteria for both Austria and Turkey were (a) age between 15 and 25 years, (b) residence in either country, and (c) sufficient German or Turkish language skills to understand the survey and provide informed consent. In total, 3665 individuals participated in the study, though we only included complete responses. While the first survey was conducted at the height of the pandemic (T1), the second survey was conducted at the beginning of the second wave (T2). We compared data from both waves, examining mental health, psychological well-being, and experiences, as well as subjective fears and cognitions related to the pandemic, between migrants and non-migrant (native) populations in both countries. Participants were split into four groups based on their self-reported backgrounds (Austrian natives, migrants living in Austria who are independent of citizenship status (Austrian migrants), Turkish natives, and migrants living in Turkey who are independent of citizenship status (Turkish migrants) which were first assessed separately (cross sectionally) at the two time points, as due to data safety restrictions, it was not possible to provide follow-up of the same (identical) individuals in both T1 to T2. We did not distinguish by time living in the country or by participants being a member of the first or second generation in the migrant groups, as we expected these additional factors to overburden the already complex structure of the groups included. For T2, the same recruitment methods were used, and our results confirmed a similar group composition to T1 was obtained, as expected.

We used the validated German [28] and Turkish [29] versions of the “Psychological General Well-being” index (PGWB) of DuPuy et al. (1984) [30], which consists of 22 items on 6-point Likert scales, divided into 6 subscales: anxiety, depressed mood, positive well-being, self-control, general health, and vitality. The PGWB captures the general well-being during the last month, but for this study, we extended the query to refer to the last two months to cover the quarantine time at the outset of the pandemic. Importantly, and in line with the manual, all subscales of the PGWB are scored in a way that higher values indicate greater well-being (i.e., a higher score in the anxiety and depression subscales means fewer indications of anxiety and depression). The psychological impact of the COVID-19 pandemic was further measured using items to assess sociodemographic data and individual experiences and changes during the COVID-19 pandemic and quarantine periods. These items included the deterioration of mental health because of the COVID-19 pandemic (response options: 1 = improved, 2 = deteriorated, 3 = unchanged), a proxy item for socioeconomic status (financial problems because of COVID-19 pandemic (1 = yes, 0 = no), and COVID-19-related conditions: ruminations about COVID-19, fear of being infected, fear relating to infection of a family member, belief that pandemic restriction measures were exaggerated, and estimated severity of COVID-19 risks (all on 5-point Likert scales, ranging from 1 = never to 5 = always). The same questionnaires were used at both time points. All study participants provided electronic informed consent with the agreement of their parents before starting the online survey. The structured online survey took approximately 10 min to complete, while only data with a complete set of responses were included.

The study was conducted in accordance with the Declaration of Helsinki, and the protocol was approved by the Ethics Commission of the Medical University of Vienna (Protocol Number: EK 1488/2020).

### Statistical Analysis

Data were analyzed using R version 4.0.3 [31]. Multilevel models (MLMs) were created to compare the four study groups at two sampling times simultaneously while taking the variability of the country in which participants were assessed (i.e., Austria or Turkey) into account (i.e., random intercept models). MLMs were fitted using the lm4 package [32] with *p*-values supplied by the lmerTest package [33]. For each of the outcomes (i.e., deterioration of mental health, subscales of the PGWB, and subscales of the COVID-19 questionnaire), we fitted a random intercept model In which “country” denotes the random intercept (Austria, Turkey) and “group” the fixed effect (Austrian native, migrant resident in Austria, Turkish native, migrant resident in Turkey). Our model also incorporated the potential confounders of gender, age, and financial problems. Interactions of group–gender and wave–gender were analyzed, followed by simple effects analyses when significant. The model was specified for all outcomes as follows:

(1) $$\begin{array}{cc} Outcome_{ij}= & \gamma_{00}+\gamma_{01}\left(Country{\;}_{j}\right)+\gamma_{10}\left(Wave_{j}\right)\\ & +\gamma_{20}\left(Group_{j}\right)+\gamma_{30}\left(Gender_{j}\right)+\gamma_{40}\left(Age_{j}\right)\\ & +\gamma_{50}\left(Financial\;Problems_{j}\right)+\gamma_{60}\left(Group_{j}\right)\\ & *\left(Gender_{j}\right)+\gamma_{70}\left(Wave_{j}\right)*\left(Group_{j}\right)+\upsilon_{0j}+e_{ij} \end{array}$$

## 3. Results

### 3.1. Participants

In total, we analyzed the data of N = 3665 participants that fit inclusion criteria and gave informed consent as outlined above. N = 1280 were assessed at time T1 and N = 2385 were assessed at time T2. Overall, n = 1056 Austrian natives, n = 380 Austria migrants, n = 1704 Turkish natives, and n = 525 Turkish migrants participated in this study. Of the Austrian migrants, 42% (n = 158) were born in Austria, while 58% (n = 222) were second-generation migrants (i.e., at least one parent was born outside of Austria). Of the first-generation migrants in the Austrian sample, 23% (n = 35) were born in Turkey, 20% (n = 32) in former Yugoslavian countries, and the remainder were born in a wide array of 35 countries. In the Turkish migrant sample, 2% (n = 11) were first-generation migrants (n = 5 born in Germany, and the remainder born in six other countries), while 98% (n = 514) were second-generation migrants. We assume that most of the participants at T1 also filled out the follow-up survey, in addition to new attendees taking part in the study at T2. In all four groups, more participants reported a decrease in mental health status and financial problems at time T2. See Table 1 for detailed sample characteristics, including age and gender distribution, across all four groups at the two time points.

### 3.2. Effects of the COVID-19 Pandemic on Mental Health and Psychological Well-Being

Detailed analyses of the decrease in mental health (our one-item indicator) revealed that overall, a significantly large group of participants reported a decrease in mental health from wave 1 to wave 2 (b = 0.06, *p* < 0.023), and this association was higher when financial problems were reported at any time (b = 0.12, *p* < 0.001). Age was not associated with the decrease in reported mental health (*p* = 0.703). Females in both the groups of Turkish natives (b = −0.21, *p* < 0.001) and migrant resident in Turkey (b = −0.25, *p* < 0.001) reported a higher proportion of decreased mental health. This was not observed in either Austrian natives or in migrants living in Austria, (ps > 0.058). Regarding changes from wave 1 to wave 2, simple effects analyses revealed that Turkish migrants reported the highest decrease in mental health status (b = 0.28 *p* < 0.001), followed by Turkish natives (b = 0.22, *p* < 0.001), Austrian migrants (b = 0.16, *p* = 0.001), and native Austrians (b = 0.06, *p* = 0.023), respectively.

**Figure 1:**
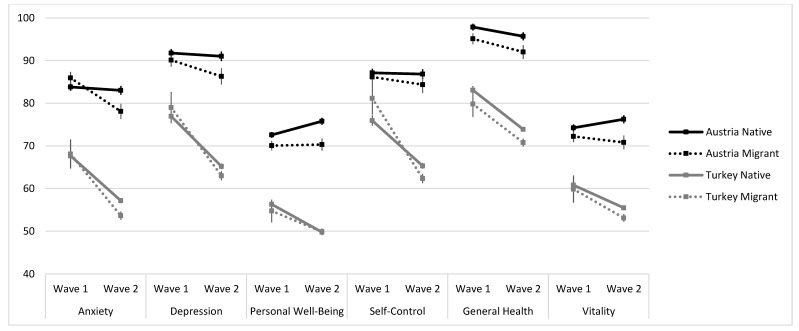
Subscales of the PGWB for the four groups, separated by wave (M and SEM).

The analyses for the subscales of the PGWB are reported in Table 2 and depicted in Figure 1. As evident in Figure 1, a decrease in all subscales can be seen across almost all four groups of young people from wave 1 to wave 2. Significant wave–group interactions (with Austrian natives coded as the reference category) showed that Austrian migrants reported an increase in symptoms (i.e., lower values) only in the subscale measuring anxiety. Both Turkish natives and Turkish migrants had a higher decrease in all subscales of the PWGB from wave 1 to wave 2 when compared with Austrian natives. Simple effects analyses furthermore revealed that Austrian natives did not report any changes in symptoms in the subscales and even showed an increase in personal well-being from wave 1 to wave 2 (b = 3.20, *p* = 0.002). Female Austrian migrants showed lower values in the subscales of personal well-being, self-control, and general health, whereas females living in Turkey (both natives and migrants) reported lower values across all subscales of the PGWB (ps < 0.047). Financial problems were associated with lower values in all subscales and across all groups (all ps < 0.001). Age was positively associated with subscales of depression, personal well-being, self-control, and vitality (ps < 0.006), indicating that older participants reported a lower symptom severity in these four domains. However, age was negatively associated with general health (*p* = 0.011) and not associated with anxiety (=0.164).

Additional exploratory analyses of all mental health outcomes with the predictor of migrant status (i.e., first or second-generation) in the two migrant groups revealed no significant associations with either the one-item mental health indicator (*p* = 0.900) or any of the PWB subscales (ps > 0.551). Our reported associations in the migrant subsamples were therefore not associated with the country of birth of participants.

### 3.3. Differences Regarding COVID-19-Related Cognitions

**Figure 2:**
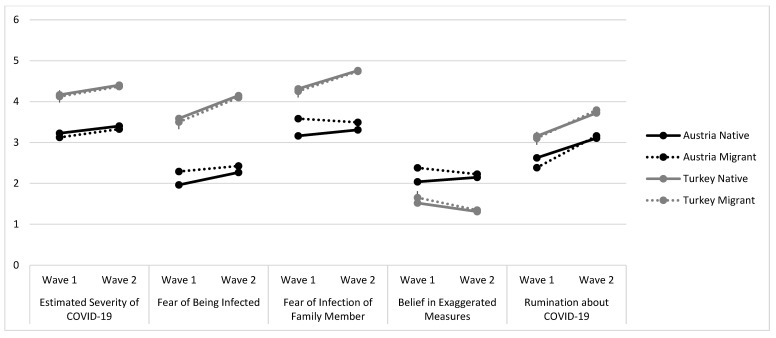
Subscales of the COVID-19-related cognitions for the four groups, separated by wave (M and SEM).

The analyses for the subscales of the questionnaire pertaining to cognitions and opinions related to the pandemic are depicted in Table 3 and Figure 2. Again, as evident in Figure 2, an increase in all subscales can be seen across almost all four groups of young people from wave 1 to wave 2. The main effects of time (i.e., wave 2 compared with wave 1) clearly showed that as the pandemic progressed, the estimated severity of COVID-19 infection (b = 0.18, *p* = 0.003), the fear of the individual being infected (b = 0.30, *p* < 0.001), and the fear of the infection of a family member (b = 0.15, *p* = 0.011) were rated higher across all four groups.

Ruminations about COVID-19 also increased across all four groups (b = 0.49, *p* < 0.001). Measures against the pandemic, however, were not rated differently at wave 2 compared with that at wave 1 (main effect *p* = 0.051). Compared with Austrian natives, migrants in Austria reported a lower fear of infection of a family member at wave 2 (compared to wave 1), a lower proportion reported belief of measures to be exaggerated, and more had pandemic-related ruminations. The group of Turkish natives had an increase in the fear of being infected, from wave 1 to wave 2, an increase in the fear of infection of a family member, and a decrease in the belief that measures might be exaggerated (ps < 0.007)—but no differences were found regarding rumination (*p* = 0.672). Turkish migrants reported a higher mean fear of infection of a family member (*p* = 0.042) and lower beliefs that measures against the pandemic might be exaggerated (*p* = 0.018). Simple effects of Austrian natives (i.e., the reference group) showed that they also—over time—reported an increase in the fear of being infected (b = 0.30, *p* < 0.001) and an increase in ruminations (b = 0.49, *p* < 0. 001). Female Austrian migrants showed lower values in the subscales for severity, fear of infection of a family member, and ruminations, whereas females living in Turkey (both natives and migrants) reported lower values across all subscales of the COVID-19 questionnaire. We found that regardless of gender, across all four groups financial problems were significantly associated with lower ratings of all items of the questionnaire except for the estimated severity of COVID-19 (*p* = 0.059). Furthermore, age was associated with higher ratings of estimated severity of COVID-19, fear of being infected, and pandemic-related ruminations (ps < 0.001).

Similar to the analyses of mental health outcomes, we ran additional exploratory analyses with the predictor of migrant status (i.e., first or second-generation) for COVID-19-related cognitions. Migrant status was not associated with any of the COVID-19-related cognitions (ps > 0.261).

## 4. Discussion

The results of this study show that the mental health status of all study groups in Austria and Turkey declined between the height of wave 1 (T1) and the early days of wave 2 (T2) of the pandemic. Though there may have been an expected improvement of self-reported mental health at T2 due to recovery time, reduced lockdown restrictions immediately preceding T2, and better preparation and developed coping mechanisms, the self-reported mental health status of young people deteriorated, as overall, more participants reported reduced mental health from wave 1 to wave 2, especially people with financial problems. However, females and migrants in the Turkish sample reported a stronger deterioration of mental health from wave 1 to wave 2 when compared with other groups. Previous studies [34,35,36] have demonstrated that females are more vulnerable to developing mental disorders during this pandemic, as well as more commonly having depressive symptoms [36,37,38] and anxiety [39,40] when compared with males. As in many other countries, migrants in Turkey generally have a lower socioeconomic status and face poverty more frequently when compared with native (non-migrant) populations [41,42], therefore, the financial crisis caused by the COVID-19 pandemic may have affected them more severely. The outbreak of COVID-19 also affected economic activities such as foreign trade and tourism, which were greatly restricted during the pandemic for several months, with a disproportionate impact on economically weaker groups in Turkey, compared with Austria. Unfortunately, the restrictions associated with the COVID-19 pandemic have led to an economic crisis in Turkey [43]. The comparison of PGWB from wave 1 to wave 2 showed a decrease in all subscales (depression, personal well-being, self-control, general health, and vitality). However, among migrants living in Austria, anxiety levels increased from wave 1 to the early part of wave 2. We suppose that the increased risk of infection and the more common adverse outcomes of COVID-19 in minority groups, compared with the native population, may have led to increased anxiety in migrants [18,19]. In the Turkish sample, natives and migrants had a more pronounced deterioration in all subscales of the PWGB from T1 (wave 1) to T2 (onset of wave 2) when compared with Austrian natives. On the contrary, Austrian natives reported quite similar symptoms in the subscales of PGWB from wave 1 to wave 2, even though their personal well-being improved. While female Austrian migrants reported a worsening in the subscales of personal well-being, self-control, and general health, females in the Turkish sample showed a deterioration in all subscales of the PGWB. Similarly, in all study groups, financial problems were associated with lower values in all subscales of the PGWB. In line with our results, diverse studies have demonstrated that females, migrants, and people with low socio-economic status are especially vulnerable to the adverse outcomes of the COVID-19 pandemic [21,35,44], which may probably have led to decreased well-being among these groups.

In the questionnaire regarding COVID-19-related cognitions, native and migrant females in Turkey reported lower values in all subscales over time. In contrast, female migrants living in Austria showed lower values in only three subscales (estimated severity of COVID-19 infection, fear of infection of a family member, and ruminations). During the course of the pandemic and the resulting lockdown measures, all four study groups were significantly more apprehensive about COVID-19, as their attitudes toward the severity of COVID-19 infection, fear of being infected themselves, or of a family member were higher during wave 2, compared with wave 1. Similarly, in all four study groups, ruminations about COVID-19 significantly increased between the two waves. Our results, therefore, indicate a higher risk of psychological problems and need of support during wave 2 instead of increased resilience and coping.

To the best of our knowledge, there is no existing study that explicitly compared two countries with different pandemic control strategies, while considering different backgrounds in domestic and cultural values. In Turkey, the measures that were set to overcome the COVID-19 pandemic were relatively stringent when compared with those in Austria. In Turkey, youths up to 20 years had a mandatory 24 h curfew for several months, as well as a general ban on people going out during the weekend. In Austria, stringent curfew measures limiting people to their houses were not effectively implemented and controlled, even during the strictest regulatory periods. Though the measures were not as strict as those in Turkey, Austrian natives were still the only group to report that the measures addressing COVID-19 were exaggerated. In contrast to the native Austrians, migrants in Austria did not view the measures as excessive. We suppose that migrants living in Austria mostly originate from low- and middle-income countries, where collectivism and respect for intervention by authorities in everyday life are more common (e.g., Turkey, Iran, Iraq, Afghanistan, Syria, etc.), than in Austria, where the culture is more individualistic (or particularistic). Therefore, they might be more open to unquestioningly accepting restrictions and consequently did not perceive the measures associated with the pandemic as excessive. Although in Turkey, the restrictions associated with the COVID-19 pandemic were much stricter, both natives and migrants did not assess the measures to be too harsh.

It might be noted that in societies that place an emphasis on the individual and their sovereign rights, any temporary suspension of those rights or privileges such as freedom of movement or association would require an appropriate justification. Aspects of these restrictions that must be addressed include (a) expected duration, (b) strict justification openly communicated to the public and legal system, (c) having no possible alternatives, and (d) in proportion to the emergent need from catastrophes such as a global pandemic.

Lockdown measures in Turkey apparently did not improve the fear of becoming infected. Turkish natives had an increased fear of infection of themselves or a family member from wave 1 to wave 2. This result may be due to the generally high rate of infection and mortality in Turkey [45], despite fairly stringent lockdown restrictions.

## 5. Limitations of Our Study

A potential limitation of this study’s conclusions is that despite the relatively large sample, an anonymous online survey may lead to a biased sample reflecting the internet skills of the user groups in question. Furthermore, due to privacy concerns, we could not follow up the same exact participants from T1 to T2 and could only use a similar group to explore the impact of the course of time and the pandemics on migrant and non-migrant populations. We further had to limit the definition of subgroups, not making a distinction between migrant groups from different cultures, or between first- and second-generation migrants. These factors will require further research and an even larger sample than the already sizeable one used in this study. Our results also could only reflect data from two countries, one with higher income and one with lower average income, as well as with different, more specific cultural, economic, and other social factors that could lead to different results in other countries. Still, we believe that our data can be used as a good indicator of possible risk factors and problems in both high- and low-economy countries. As similar groups, if not the same individuals, were used to compare T1 during the height of wave 1 and T2 early in wave 2, results and our conclusions might have been influenced by differences in group composition as to age, gender, or socioeconomic status and might also have to be followed up by further research.

## 6. Conclusions

Due to the pandemic and its associated restrictions, the psychological stress on children, adolescents, and families was shown to increase over time [9] and did not lead to better adaptation, which are findings that were confirmed by our data. It is certain that some forms of restrictions were required to slow the COVID-19 pandemic, but we should also examine the potential adverse impact on the mental health of young individuals: “collateral” damage of both the pandemic and restrictions. Necessary restrictions must be justified and weighed against the negative impacts and limitations on individual freedoms. During the pandemic, young people were especially vulnerable to psychological problems [13,14,46], especially those with preexisting mental health problems [21]. Consequently, these minors must be seen as more vulnerable, with an increased need for support and therapy [47]. This implies that in Europe a strong “recovery plan” for children and adolescents to cope with the mental health impact of current or future pandemics should be carefully prepared with consideration to minority needs and vulnerable groups and should be implemented as soon as possible. This recovery plan should be able to repair psychological problems among minors to avoid the further manifestation of mental health problems as well as other adverse consequences such as academic deficiency, unemployment, and poverty [47].

The present cross-national comparison of Turkey and Austria suggests that an “authoritarian” approach to lockdowns may not be the most effective counter to a pandemic, as Turkey had significantly more stringent measures than Austria but still had a higher rate of infection and mortality [45]. Austria had more moderate restrictions, but a relatively lower number of cases, which implies that there may be other contributing factors to determining the best strategy to limit a pandemic.

During the COVID-19 pandemic, UN agencies emphasized the human rights of migrants and refugees and the need to establish pandemic restrictions that still respect these rights [48]. The COVID-19 pandemic has clearly highlighted the inequalities in society, and the need for intersectional policies and strategies to improve the health and living conditions of migrants. Countries with a high number of migrants should especially be able to ensure basic human rights for the migrant populations [44]. They must find a balance between necessary restrictions to address a catastrophic pandemic while finding ways to protect against unintended side effects of those same restrictions. Only then can we limit the harm from both the pandemic and its “collateral” damage on society, and especially vulnerable groups.

The results of this study can help understand the role of both culture and socioeconomic factors such as a financial background in identifying at-risk groups during similar pandemics or other catastrophes. Identifying at-risk groups is necessary to develop adequate specialized mental health services for the target groups. Therefore, the present results can be considered the first step in this research. Furthermore, longitudinal studies, in particular, are needed for a detailed examination of the impact of the COVID-19 pandemic’s progression on the mental health of individuals.

## Figures and Tables

**Table 1:** Description of the sample at the two time points: wave 1 = 22 May–19 June; wave 2 = 11 September–23 October.

	Wave 1 (n = 1280)			Wave 2 (n = 2385)		
	Age M (SD)	Decrease in Mental Health (% (n) Yes)	Financial Problems (% (n) Yes)	Age M (SD)	Decrease in Mental Health (% (n) Yes)	Financial Problems (% (n) Yes)
**Sample Austria (n = 1436**)	19.98 (3.31)	29 (n = 248)	14 (n = 121)	20.42 (3.60)	38 (n = 219)	13 (n = 74)
Austrian Natives (n = 1056)	20.26 (3.43)	31 (n = 193)	12 (n = 76)	20.50 (3.57)	37 (n = 160)	12 (n = 115)
Male (n = 314)	19.64 (3.47)	21 (n = 38)	11 (n = 20)	20.57 (3.70)	24 (n = 32)	7 (n = 9)
Female (n = 731)	20.54 (3.39)	35 (n = 152)	12 (n = 54)	20.47 (3.53)	43 (n = 126)	13 (n = 38)
Migrants in Austria (n = 380)	19.23 (2.83)	24 (n = 55)	19 (n = 45)	20.18 (3.69)	40 (n = 59)	16 (n = 24)
Male (n = 143)	19.29 (2.76)	19 (n = 19)	18 (n = 18)	20.25 (3.85)	39 (n = 17)	11 (n = 5)
Female (n = 235)	19.19 (2.91)	27 (n = 35)	20 (n = 26)	20.15 (3.66)	40 (n = 41)	18 (n = 19)
**Sample Turkey (n = 2229)**	21.20 (3.32)	51 (n = 216)	40 (n = 171)	21.95 (2.87)	74 (n = 1343)	52 (n = 941)
Turkish Natives (n = 1704)	21.14 (3.30)	51 (n =197)	38 (n = 150)	21.88 (2.90)	74 (n = 975)	53 (n = 692)
Male (n = 1132)	21.13 (3.20)	53 (n = 149)	39 (n = 111)	21.98 (2.76)	76 (n = 647)	53 (n = 450)
Female (n = 565)	21.12 (3.57)	46 (n = 47)	37 (n = 38)	21.71 (3.10)	70 (n = 326)	52 (n = 240)
Migrants in Turkey (n = 525)	21.82 (3.49)	48 (n = 19)	53 (n = 252)	22.15 (2.79)	76 (n = 368)	51 (n = 249)
Male (n = 331)	20.75 (3.30)	54 (n = 13)	38 (n = 9)	22.05 (2.70)	79 (n = 243)	54 (n = 167)
Female (n = 194)	23.44 (3.22)	38 (n = 6)	75 (n = 12)	22.33 (3.00)	70 (n = 125)	46 (n = 82)

Note. n = 20 participants indicated a diverse gender (n = 13 at wave 1; n = 7 at wave 2).

**Table 2:** Results of multilevel models of the subscales of the PGWB.

	Anxiety		Depression		Personal Well-Being		Self-Control		General Health		Vitality	
Predictors	Estimates	*p*	Estimates	*p*	Estimates	*p*	Estimates	*p*	Estimates	*p*	Estimates	*p*
(Intercept)	88.48	<0.001	87.14	<0.001	73.06	<0.001	86.07	<0.001	108.66	<0.001	73.75	<0.001
wave 2	−0.80	0.538	−0.91	0.519	3.14	0.002	−0.52	0.724	−2.22	0.064	1.92	0.115
Austria Migrant	−0.41	0.857	−2.48	0.310	−5.24	0.004	−5.60	0.029	−7.85	<0.001	−4.01	0.059
Turkey Native	−22.35	<0.001	−20.18	0.203	−20.26	0.321	−19.18	<0.001	−18.85	0.447	−16.91	0.246
Turkey Migrant	−21.37	<0.001	−16.95	0.296	−22.20	0.280	−12.90	0.036	−21.86	0.381	−17.70	0.234
Female	−9.17	<0.001	−7.79	<0.001	−6.46	<0.001	−10.47	<0.001	−6.61	<0.001	−5.75	<0.001
Age	0.15	0.164	0.58	<0.001	0.24	0.006	0.49	<0.001	−0.26	0.011	0.28	0.006
Financial Problems	−9.25	<0.001	−11.38	<0.001	−5.70	<0.001	−8.98	<0.001	−6.94	<0.001	−8.50	<0.001
Austria Migrant * Female	3.50	0.181	1.83	0.518	4.29	0.040	7.28	0.014	7.62	0.002	3.53	0.150
Turkey Native * Female	16.41	<0.001	15.43	<0.001	8.82	<0.001	18.72	<0.001	11.63	<0.001	10.46	<0.001
Turkey Migrant * Female	15.44	<0.001	12.92	<0.001	10.49	<0.001	15.56	<0.001	12.25	<0.001	10.98	<0.001
Wave 2 * Austria Migrant	−6.77	0.008	−3.14	0.256	−2.86	0.159	−1.86	0.520	−1.13	0.631	−3.46	0.149
Wave 2 * Turkey Native	−9.20	<0.001	−10.28	<0.001	−9.29	<0.001	−9.92	<0.001	−6.18	<0.001	−6.68	<0.001
Wave 2 * Turkey Migrant	−13.60	<0.001	−15.24	<0.001	−8.05	0.005	−18.39	<0.001	−6.64	0.048	−8.71	0.011
**Random Effects**												
σ^2^	429.83		498.70		270.83		547.54		362.27		376.11	
τ_00_	424.05 _Country_		123.80 _Country_		207.18 _Country_		10.52 _Country_		305.49 _Country_		104.75 _Country_	
ICC	0.50		0.20		0.43		0.02		0.46		0.22	
N	2 _Country_		2 _Country_		2 _Country_		2 _Country_		2 _Country_		2 _Country_	
Observations	3645		3645		3645		3645		3645		3645	
Marginal R^2^/Conditional R^2^	0.313/NA		0.237/0.389		0.217/0.556		0.205/0.220		0.170/0.550		0.181/0.359	

Note. All factorial variables were dummy coded with the reference categories: wave 2 vs. wave 1; Austria migrant, Turkey native, and Turkey migrant vs. Austria native; female vs. male; financial problems indicated vs. not indicated. σ^2^ = fixed-effect variance, τ_00_ = between-subject variance, ICC = intraclass correlation.

**Table 3:** Results of multilevel models of the subscales of the COVID-19-related cognitions questionnaire.

	Estimated Severity of COVID-19		Fear of Being Infected		Fear of Infection of a Family Member		Belief in Exaggerated Measures		Rumination about COVID-19	
Predictors	Estimates	*p*	Estimates	*p*	Estimates	*p*	Estimates	*p*	Estimates	*p*
(Intercept)	2.57	<0.001	1.28	0.565	3.02	0.011	2.21	<0.001	1.51	<0.001
wave 2	0.18	0.003	0.30	<0.001	0.15	0.011	0.12	0.050	0.49	<0.001
Austria Migrant	0.17	0.093	0.41	0.001	0.61	<0.001	0.44	<0.001	0.01	0.897
Turkey Native	1.18	<0.001	1.82	0.563	1.44	0.390	−0.60	0.114	0.75	0.175
Turkey Migrant	1.21	<0.001	1.77	0.573	1.41	0.402	−0.56	0.175	0.77	0.181
Female	0.34	<0.001	0.29	<0.001	0.44	<0.001	−0.01	0.834	0.39	<0.001
Age	0.02	<0.001	0.02	<0.001	−0.01	0.071	−0.01	0.091	0.04	<0.001
Financial Problems	0.07	0.059	0.16	<0.001	0.12	0.001	0.07	0.049	0.13	<0.001
Austria Migrant * Female	−0.38	0.002	−0.05	0.694	−0.26	0.032	−0.18	0.146	−0.28	0.022
Turkey Native * Female	−0.49	<0.001	−0.53	<0.001	−0.48	<0.001	0.27	0.001	−0.46	<0.001
Turkey Migrant * Female	−0.67	<0.001	−0.63	<0.001	−0.56	<0.001	0.39	<0.001	−0.73	<0.001
Wave 2 * Austria Migrant	0.03	0.816	−0.20	0.132	−0.26	0.026	−0.25	0.040	0.25	0.041
Wave 2 * Turkey Native	0.05	0.526	0.25	0.007	0.29	<0.001	−0.36	<0.001	0.04	0.672
Wave 2 * Turkey Migrant	0.05	0.755	0.28	0.142	0.34	0.042	−0.41	0.018	0.18	0.305
**Random Effects**										
σ^2^	0.89		1.18		0.90		0.96		0.95	
τ_00_	0.02 _Country_		4.94 _Country_		1.40 _Country_		0.07 _Country_		0.15 _Country_	
ICC	0.03		0.81		0.61		0.07		0.14	
N	2 _Country_		2 _Country_		2 _Country_		2 _Country_		2 _Country_	
Observations	3645		3645		3645		3645		3645	
Marginal R^2^/Conditional R^2^	0.249/0.268		0.129/0.832		0.175/0.678		0.142/0.200		0.197/0.307	

Note. All factorial variables were dummy coded with the reference categories: wave 2 vs. wave 1; Austria migrant, Turkey native, and Turkey migrant vs. Austria native; female vs. male; financial problems indicated vs. not indicated. σ^2^ = fixed-effect variance, τ_00_ = between-subject variance, ICC = intraclass correlation.

## Data Availability

The data are not publicly available due to privacy.
